# Particulate Air Pollution and Fasting Blood Glucose in Nondiabetic Individuals: Associations and Epigenetic Mediation in the Normative Aging Study, 2000–2011

**DOI:** 10.1289/EHP183

**Published:** 2016-05-26

**Authors:** Cheng Peng, Marie-Abele C. Bind, Elena Colicino, Itai Kloog, Hyang-Min Byun, Laura Cantone, Letizia Trevisi, Jia Zhong, Kasey Brennan, Alexandra E. Dereix, Pantel S. Vokonas, Brent A. Coull, Joel D. Schwartz, Andrea A. Baccarelli

**Affiliations:** 1 Department of Environmental Health, Harvard T.H. Chan School of Public Health, Boston, Massachusetts, USA; 2 Department of Statistics, Harvard University, Cambridge, Massachusetts, USA; 3 Department of Geography and Environmental Development, Ben-Gurion University of the Negev, Beersheba, Israel; 4 Human Nutrition Research Center, Institute of Cellular Medicine, Newcastle University, Newcastle, United Kingdom; 5 Molecular Epidemiology and Environmental Epigenetics Laboratory, Department of Clinical Sciences and Community Health, University of Milan, Milan, Italy; 6 VA Normative Aging Study, Veterans Affairs Boston Healthcare System, Boston, Massachusetts, USA; 7 Department of Medicine, Boston University School of Medicine, Boston, Massachusetts, USA; 8 Department of Biostatistics, Harvard T.H. Chan School of Public Health, Boston, Massachusetts, USA; 9 Channing Laboratory, Harvard Medical School, Boston, Massachusetts, USA

## Abstract

**Background:**

Among nondiabetic individuals, higher fasting blood glucose (FBG) independently predicts diabetes risk, cardiovascular disease, and dementia. Ambient PM2.5 (particulate matter with aerodynamic diameter ≤ 2.5 μm) is an emerging determinant of glucose dysregulation. PM2.5 effects and mechanisms are understudied among nondiabetic individuals.

**Objectives:**

Our goals were to investigate whether PM2.5 is associated with an increase in FBG and to explore potential mediating roles of epigenetic gene regulation.

**Methods:**

In 551 nondiabetic participants in the Normative Aging Study, we measured FBG, and DNA methylation of four inflammatory genes (IFN-γ, IL-6, ICAM-1, and TLR-2), up to four times between 2000 and 2011 (median = 2). We estimated short- and medium-term (1-, 7-, and 28-day preceding each clinical visit) ambient PM2.5 at each participant’s address using a validated hybrid land-use regression satellite-based model. We fitted covariate-adjusted regression models accounting for repeated measures.

**Results:**

Mean FBG was 99.8 mg/dL (SD = 10.7), 18% of the participants had impaired fasting glucose (IFG; i.e., 100–125 mg/dL FBG) at first visit. Interquartile increases in 1-, 7-, and 28-day PM2.5 were associated with 0.57 mg/dL (95% CI: 0.02, 1.11, p = 0.04), 1.02 mg/dL (95% CI: 0.41, 1.63, p = 0.001), and 0.89 mg/dL (95% CI: 0.32, 1.47, p = 0.003) higher FBG, respectively. The same PM2.5 metrics were associated with 13% (95% CI: –3%, 33%, p = 0.12), 27% (95% CI: 6%, 52%, p = 0.01) and 32% (95% CI: 10%, 58%, p = 0.003) higher odds of IFG, respectively. PM2.5 was negatively correlated with ICAM-1 methylation (p = 0.01), but not with other genes. Mediation analysis estimated that ICAM-1 methylation mediated 9% of the association of 28-day PM2.5 with FBG.

**Conclusions:**

Among nondiabetics, short- and medium-term PM2.5 were associated with higher FBG. Mediation analysis indicated that part of this association was mediated by ICAM-1 promoter methylation.

**Citation:**

Peng C, Bind MA, Colicino E, Kloog I, Byun HM, Cantone L, Trevisi L, Zhong J, Brennan K, Dereix AE, Vokonas PS, Coull BA, Schwartz JD, Baccarelli AA. 2016. Particulate air pollution and fasting blood glucose in nondiabetic individuals: associations and epigenetic mediation in the Normative Aging Study, 2000–2011. Environ Health Perspect 124:1715–1721; http://dx.doi.org/10.1289/EHP183

## Introduction

Clinically diagnosed diabetes is preceded by a long latent period of abnormal glucose metabolism (American Diabetes Association 2012). In the asymptomatic, nondiabetic range of glycemia (< 126 mg/dL) (American Diabetes Association 2012), increased fasting blood glucose (FBG) levels are already independently associated with the development of diabetes (Tirosh et al. 2005), cardiovascular disease (Duckworth 2001; Levitan et al. 2004; Sarwar et al. 2010), and dementia (Crane et al. 2013). FBG variations in this range are often underappreciated and their potential determinants, especially those not directly related to lifestyle, are understudied. Ambient particulate matter (PM) pollution has recently been suggested as an emerging risk factor for metabolic disorders including impaired glucose regulation (Esposito et al. 2016; Rajagopalan and Brook 2012). Cross-sectional and longitudinal studies have revealed that PM is associated with increased risk of diabetes (Andersen et al. 2012; Brook et al. 2008; Puett et al. 2011) and with higher levels of markers of insulin resistance (Kelishadi et al. 2009). However, the relationship between PM and glycemia in the nondiabetic range has yet to be studied.

The underlying mechanisms linking PM and abnormal glucose regulation are also not fully understood. Inflammation is central in both PM-associated responses and the pathogenesis of glucose dysregulation. Evidence from previous studies has linked exposures to ambient PM with lower DNA methylation in inflammatory genes (Bind et al. 2012, 2014). DNA methylation, through the addition of a methyl group to the 5C position of cytosine in the CpG dinucleotide sequence, is a well-studied epigenetic modification that usually silences gene expression (Newell-Price et al. 2000). Conversely, lower or no methylation has been associated with upregulated gene expression. Lower global methylation content across the human epigenome has been associated with hyperglycemia and the up-regulation of inflammatory genes in peripheral leukocytes from patients with type 2 diabetes mellitus (T2DM) (Luttmer et al. 2013; Nilsson et al. 2014). Yet, the role of methylation of specific genes related to inflammation in mediating the effects of PM on FBG has not been investigated.

In a repeated measure study of older men in the Greater Boston Area, we investigated the association between ambient PM_2.5_ (PM with aerodynamic diameter ≤ 2.5 μm) concentrations at the participants’ addresses—estimated over different time windows up to 1 month before the visit—and FBG levels among nondiabetic participants. Using recently developed repeated measure mediation analysis, we further examined whether and to what extent PM_2.5_ increased FBG through changes in blood leukocyte methylation in candidate inflammatory genes. We examined methylation of inflammatory cytokines [interferon gamma (*IFN-*γ) and interleukin-6 (*IL-6*)], intercellular adhesion molecule-1 (*ICAM-1*), and Toll-like receptor 2 (*TLR-2*). We hypothesized that higher PM_2.5_ levels associated with increased FBG, and part of this association was mediated through methylation of inflammatory genes.

## Materials and Methods

### Study Population

The Normative Aging Study is a prospective longitudinal cohort established in 1963 by the U.S. Veterans Administration in the Greater Boston area (Power et al. 2011). Briefly, participants underwent examinations every 3–5 years. Self-administered questionnaires were collected at each visit providing information on sociodemographic characteristics, medical history, medications, and lifestyle. Blood samples were collected at each clinical visit, after an overnight fast and smoking abstinence (see Figure S2). Starting from year 2000, estimated concentrations of PM_2.5_ were obtained from a hybrid spatiotemporal prediction model, as described in the next section. A total of 656 participants had complete information on PM_2.5_ measurements from the prediction model, FBG, and blood leukocyte DNA methylation for at least one and up to four visits between 2000 and 2011 (median = 2, IQR = 1). We excluded 105 participants who, at their first visit, were clinically diabetic (FBG ≥ 126 mg/dL at the visit) and were taking diabetes medications. Therefore, our final study population included 551 participants. Fifty-two participants were diagnosed with diabetes during subsequent visits: For these individuals, we retained observations from the visit(s) before they were diagnosed with diabetes. One hundred and eighty-six participants came to just one clinical visit, 163 participants came to two clinical visits, and 202 participants came to three or more clinical visits. Participants provided signed informed consent at each visit. The study was approved by the Institutional Review Boards of the participating institutions.

### Air Pollution and Temperature

We estimated PM_2.5_ concentrations at each participant’s residential address using a hybrid land use regression and satellite-based model (Kloog et al. 2012; Madrigano et al. 2013). In brief, we utilized MODIS (Moderate Resolution Imaging Spectroradiometer) satellite-derived aerosol optical depth (AOD) measurements to predict daily PM_2.5_ concentration levels at a 10 km spatial resolution. Daily AOD was calibrated using ground PM_2.5_ measurements from 78 monitoring stations, land use regression and meteorological variables. To estimate PM_2.5_ daily concentrations in each grid cell, we calibrated the AOD-PM_2.5_ relationship using data from grid cells with both monitor and AOD values, using mixed models with random slopes for day and nested regions. In a later stage, we estimated exposures on days when AOD measures were not available (e.g., due to cloud cover or snow). Model performance was good with high out-of-sample 10-fold cross-validated *R* ^2^ (mean out-of-sample *R* ^2^ = 0.83 and 0.81 for days with and without available AOD data, respectively) (Kloog et al. 2011).

Temperature values were obtained through the national climatic data center. Only continuous operating stations that collected data on a daily basis were used (26 stations). Grid cells were matched to the closest weather station for meteorological variables.

### DNA Methylation Measurements

Gene-specific DNA methylation was quantified on buffy-coat DNA using bisulfite polymerase-chain-reaction pyrosequencing (Yang et al. 2004). In the Normative Aging Study, we generated pyrosequencing-based methylation data for nine genes across pathways related to oxidation, blood clotting, and inflammation (Bind et al. 2012; Lepeule et al. 2012, 2014). We chose to focus our analysis on the four inflammatory genes included in these genes, including two inflammatory cytokines (*IFN-*γ and *IL-6*), intercellular adhesion molecule 1 (*ICAM-1*), and Toll-like receptor 2 (*TLR-2*). The choice of these four inflammatory genes was aligned with previous literature on PM and inflammation, as well as subclinical inflammation and the risk of T2DM (Bind et al. 2012; Blüher et al. 2002; Herder et al. 2013; Madrigano et al. 2010; van Eeden et al. 2001; Vossoughi et al. 2014). Specifically, *IFN-*γ and *IL-6* encode for inflammatory cytokines that facilitate cell-to-cell communications in the inflammatory cascade, *ICAM-1* encodes for a glycoprotein that is often expressed on the cell surface of endothelial cells and leukocytes, ICAM-1 glycoproteins are important for cell surface adhesion, transmigration and homing of leukocytes from the circulation to the target tissue. *TLR-2* encodes for a surface receptor protein, which recognizes conservative molecular patterns and serves as first-line defense in innate immunity. DNA methylation levels were measured for each of these genes at two to five CpG sites within the promoter region except for *IL-6* methylation, which was measured within 500 base-pair downstream of the gene’s promoter region where nuclear respiratory factor-1 (NRF-1) binding sites are located (see Figure S1). The methylation of NRF-1 region is known to suppress *IL-6* gene expression (Choi et al. 2004). We calculated and used the mean level of position-specific DNA methylation for each gene because they are highly correlated and are likely to share most of the same functional complexes and traits, and we assumed that mean methylation across the promoter region reflects regional epigenetic regulation (Bind et al. 2014).

### Fasting Blood Glucose Measurement

Blood glucose levels were measured at each visit, after an overnight fast and were analyzed using the enzymatic hexokinase method. According to American Diabetes Association criteria, FBG less than 100 mg/dL corresponds to normal levels and the 100–125 mg/dL range to impaired fasting blood glucose (IFG), which in older individuals often progresses to diabetes over time. FBG larger than 125 mg/dL is defined as clinical diabetes (American Diabetes Association 2008, 2012).

### Statistical Analysis of the Main Association of PM_2.5_ Levels with FBG

We evaluated the association between PM_2.5_ levels and FBG (modeled as a continuous dependent variable) using linear mixed-effects regression with subject-specific intercepts to account for the correlation among repeated FBG measurements within the same individual. Exposure variables included averages of PM_2.5_ concentrations for 1-, 7-, and 28-day preceding each clinical visit; we considered each moving average in a separate regression model. Model estimates are expressed per interquartile range (IQR) increase in PM_2.5_ concentration. In the models, we adjusted for the following covariates selected *a priori*: age (continuous), body mass index [BMI; weight (kg)/height (m)^2^, continuous], race (white or others), regular patterns of physical activity (< 12 hr/week, ≥ 12 and < 30 hr/week, ≥ 30 hr/week), smoking status (never, former, or current smoker), cumulative pack-years of smoking (continuous), alcohol consumption (< 2 or ≥ 2 drinks/day), education level (high school diploma or less, college degree, or graduate degree), statin use (nonuser, current user), temperature (continuous), and seasonality. Seasonality was modeled using Fourier series terms cos(2π * doy/365.25) and sin(2π * doy/365.25), where doy represents day of year. We checked the linearity assumptions of the continuous covariates using cubic splines and found no deviation from linear dose response.

In Equation 1, the main regression model took the general form:

Y_ij_ = β_0_ + u_i_ + β_1_X_1ij_ + … + β_p_X_pij_ + β_PM2.5_PM_2.5_ + ε_ij_, [1]

where i corresponds to each participant, j to the visit; β_0_ to the intercept for the population mean; u_i_ to the subject-specific random intercept. β_1_X_1ij_ to β_p_X_pij_ correspond to the covariates we selected *a priori*. β_PM2.5_PM_2.5_ corresponds to PM_2.5_ levels 1-, 7-, or 28-day prior to the clinical visits, depending on the moving average used in each set of models. ε_ij_ is the within-subject error term.

In a secondary analysis, we considered a dichotomized FBG variable for impaired fasting glucose (IFG) (categorized using the 0–100 mg/dL and 100–125 mg/dL ranges) as the outcome and evaluated the association between PM_2.5_ and the odds of IFG using a logistic regression model with generalized estimating equations (GEE) and empirical variance estimates to account for repeated measurements per subject.

In Equation 2, the logistic regression model took the general form:

logit [Pr(Y_ij_ = 1)] = β_0_ + β_1_X_ij_ + … + β_p_X_ij_ + β_PM2.5_PM_2.5ij_, [2]

where i corresponds to each participant, j to the visit; β_0_ to the intercept for the population mean. β_1_X_ij_ to β_p_X_ij_ corresponds to the covariates we selected *a priori*. β_PM2.5_PM_2.5ij_ corresponds to PM_2.5_ levels 1-, 7-, and 28-day prior to the clinical visits, respectively. Y_ij_ = 0 indicates subject i is not defined as IFG at visit j; Y_ij_ = 1 indicates subject i is IFG at visit j.

To account for potential selection bias due to loss of follow-up, we repeated our analyses using inverse probability weighting. Specifically, in a logistic regression, we predicted the probability of coming to a subsequent visit based on covariates from the previous one, including age, BMI, regular patterns of physical activity, smoking status, pack-year smoked, FEV1 and FVC ratio, medication (diuretics and beta blockers), and education level.

### Statistical Analysis of DNA Methylation and Mediation Analysis

***Selection of mediators.*** We hypothesized that associations of PM_2.5_ with FBG could be mediated through changes in gene-specific methylation of inflammatory biomarkers. We considered the four inflammation genes—*IFN-*γ, *IL-6*, *ICAM-1* and *TLR-2*—separately in the mediation analysis. To approximate normality of the residuals, we used *IFN-*γ and *IL-6* on their original scale and log-transformed *ICAM-1* and *TLR-2.*

For DNA methylation of a specific gene to be considered as a potential mediator, we tested the following criteria *a*) if there was an association between exposure and mediator; and *b*) if there was an association between mediator and outcome (Baron and Kenny 1986; Valeri and Vanderweele 2013). We also examined the presence of PM-mediator interactions and found no evidence of interactions that changed FBG levels.

***Underlying assumptions.*** To obtain valid estimates of the natural indirect effects, we adjusted for potential exposure–outcome confounders (denoted as C_1_), exposure–mediator confounders (denoted as C_2_), and mediator–outcome confounders (denoted as C_3_), which included age, BMI, race, regular patterns of physical activity, smoking status, cumulative pack-years smoked, alcohol consumption, education level, statin use, temperature, seasonality, batch of methylation measurement, percentages of lymphocytes, and percentage of neutrophils. We assumed no unmeasured confounding for *a*) PM_2.5_-FBG relation, *b*) methylation-FBG relation, *c*) PM_2.5_-methylation relation, after fitting the linear mixed-effects models with subject-specific intercepts and controlling for C_1_, C_2_, and C_3_. In addition, we also assumed that no methylation–FBG confounders would be affected by PM_2.5_ exposure.

**Figure 1:**
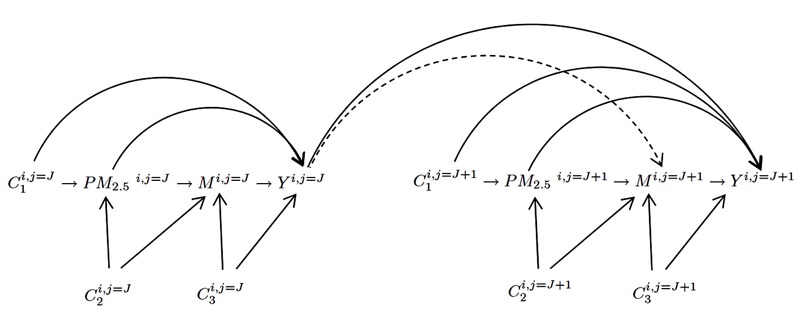
Directed acyclic graph (DAG) for mediation analysis. PM_2.5_^i,j = J^ represents air pollution exposure for ith subject prior to j = Jth visit; M^i,j = J^ represents gene-specific DNA methylation for ith subject at jth visit; Y^i,j = J^ represents fasting blood glucose (FBG) concentrations for ith subject at jth visit. C_1_^i,j^ represents exposure outcome confounders; C_2_^i,j^ represents exposure mediator confounders; C_3_^i,j^ represents mediator outcome confounders. Note: to be simplified, correlations between repeated measures of exposures (i.e., PM_2.5ij_ and PM_2.5ij + 1_), repeated measures of mediators (i.e., M_ij_ and M_ij + 1_) and repeated measures of confounders (i.e., C_ij_ and C_ij + 1_) are not shown in this DAG.

Due to the longitudinal nature of the study, changes in FBG at one visit could potentially affect gene-specific methylation at the subsequent visit [Y_ij_ → M_ij + 1_ (dotted arrow in Figure 1)]. FBG at one visit therefore may serve as a potential mediator-outcome confounder for the subsequent visit and may introduce bias in our estimates (Diggle et al. 2013). Therefore, in Equation 3, we tested the presence of an association between Y_ij_ and M_ij + 1_, to check the assumption of time-varying confounding:

M_ij + 1_ = α_0_ + u_i_ + α_1_Y_ij_ + … + α_p_X_pij_ + α_PM2.5_PM_2.5ij_ + ε_ij_, [3]

where i corresponds to each participant and j to the visit; α_0_ to the intercept for the population mean; u_i_ to the subject-specific random intercept. M_ij + 1_ corresponds to DNA methylation at subsequent visit. Y_ij_ corresponds to FBG measurement. α_1_X_1ij_ to α_p_X_pij_ correspond to the covariates we selected *a priori*. α_PM2.5_PM_2.5_ corresponds to PM_2.5_ levels 1-, 7-, and 28-day prior to the clinical visits, respectively. ε_ij_ is the within-subject error term.

***Mediation analysis.*** We fitted two linear mixed-effects models with random intercepts simultaneously, one modeling the exposure–mediator association, and one modeling the mediator–outcome association (Bauer et al. 2006; Bind et al. 2016) (Equations 4 and 5):

M_ij_ = β_0_ + u_i_ + β_1_X_1ij_ + … + β_p_X_pij_ + β_PM2.5_PM_2.5ij_ + ε_ij_, [4]

Y_ij_ = γ_0_ + g_0i_ + γ _1_X_1ij_ + … + γ _p_X_pij_ + γ _PM2.5_PM_2.5ij_ + γ _M_M_ij_ + η_ij_, [5]

where i corresponds to each participant and j to the visit; β_0_ and γ_0_ to the intercept for the population mean; u_i_ and g_0i_ to the subject-specific random intercept. β_1_X_1ij_ to β_p_X_pij_ and γ _p_X_pij_ to γ _p_X_pij_ correspond to the covariates we selected *a priori*. M_ij_ corresponds to DNA methylation and Y_ij_ corresponds to FBG measurement. β_PM2.5_PM_2.5_ corresponds to PM_2.5_ levels 1-, 7-, and 28-day prior to the clinical visits in the exposure–mediator association, and γ _M_M_ij_ corresponds to DNA methylation in the mediator-outcome association. ε_ij_ and η_ij_ are the within-subject error terms.

γ _PM2.5_ corresponds to the natural direct effect, and the natural indirect effect (also called “mediated” effect) is given by the product of β_PM2.5_ × γ _M_. The delta method was used to calculate the variance of the natural indirect effect, which corresponds to Var (γ _M_) β_PM2.5_^2^ + 2Cov(β_PM2.5_, γ _M_) β_PM2.5_ γ_M_ + Var(β_PM2.5_) γ _M_^2^. Proportion mediated is calculated as the percentage of natural indirect effect over the sum of natural direct and natural indirect effect (i.e., {[β_PM2.5_ × γ _M_/(β_PM2.5_ × γ _M_ + γ _PM2.5_)]}).

In sensitivity analysis, we tested the robustness of the study findings to the no-unmeasured confounding assumptions: *a*) we excluded participants who were current smokers to better control for residual confounding by smoking; *b*) we additionally controlled for total calorie intake and glycemic index to reduce potential mediator–outcome confounding from diet; *c*) we also restricted the analysis to participants with a C-reactive protein (CRP) level < 10 mg/L to partially remove potential effects from acute inflammation.

All analyses were conducted with SAS (version 9.3; SAS Institute Inc.), using PROC MIXED to fit the linear mixed effect models and PROC GENMOD to fit the GEE models.

## Results

### Descriptive Statistics

A total of 1,152 FBG measurements were collected from the 551 nondiabetic participants in this study. Table 1 describes participant characteristics at their first visit. Mean FBG concentration at the first visit was 99.8 ± 10.7 mg/dL (mean ± SD) and 18% of participants at the first visit had IFG (i.e., blood glucose between 100 mg/dL and 125 mg/dL). Summary statistics of PM_2.5_ and temperature during the study period are presented in Table S1.

**Table 1:** Characteristics of the Normative Aging Study participants included in the analysis, 2000–2011.

Variable	Visit 1	Visit 2	Visit 3	Visit 4
Age (years), mean ± SD	73.3 ± 6.9	75.6 ± 6.4	77.9 ± 5.9	78.5 ± 5.8
BMI (kg/m^2^), mean ± SD	27.8 ± 3.7	27.4 ± 3.7	27.1 ± 3.6	27.5 ± 3.9
Smoking status, *n* (%)
Never	165 (30%)	112 (31%)	71 (34%)	14 (33%)
Former	363 (66%)	244 (67%)	135 (64%)	1 (2%)
Current	23 (4%)	7 (2%)	5 (2%)	28 (65%)
Pack-years, mean ± SD	19.9 ± 24.7	18.7 ± 23.1	17.1 ± 21.3	16.0 ± 11.5
Race, *n* (%)
White	543 (97%)	354 (98%)	203 (96%)	42 (98%)
Other	17 (3%)	9 (2%)	8 (4%)	1 (2%)
Metabolic equivalent of task, *n* (%)
Low (≤ 12 hr/week)	353 (64%)	222 (61%)	129 (61%)	26 (60%)
Medium (12–30 hr/week)	111 (20%)	89 (25%)	47 (22%)	10 (23%)
High (≥ 30 hr/week)	87 (16%)	52 (14%)	35 (17%)	7 (16%)
Two or more drinks per day, *n* (%)	102 (19%)	69 (19%)	35 (17%)	4 (9%)
Education, *n* (%)
< 12 years	181 (33%)	119 (32%)	63 (30%)	11 (26%)
13–16 years	253 (46%)	163 (45%)	98 (46%)	24 (56%)
> 16 years	115 (21%)	81 (22%)	50 (24%)	8 (19%)
Statin use, *n* (%)	196 (36%)	180 (50%)	122 (58%)	24 (56%)
Fasting blood glucose (mg/dL)	99.8 ± 10.7	98.9 ± 10.0	99.0 ± 10.5	99.0 ± 11.5
Impaired fasting blood glucose, *n* (%)	100 (18%)	54 (15%)	38 (18%)	7 (16%)
Blood *IFN-*γ methylation, mean ± SD	84.3 ± 5.9	85.0 ± 4.8	85.3 ± 4.7	85.3 ± 5.5
Blood *IL-6* methylation, mean ± SD	43.6 ± 10.3	43.5 ± 10.2	43.8 ± 10.3	42.8 ± 11.5
Blood *ICAM* methylation, mean ± SD	4.3 ± 1.8	3.9 ± 1.2	4.4 ± 1.2	4.8 ± 1.9
Blood* TLR2* methylation, mean ± SD	3.1 ±1.3	3.0 ± 1.4	2.5 ± 1.4	1.9 ± 1.0
Note: Cohort participants with diabetes were excluded.				

### Main Association of PM_2.5_ Levels with FBG

PM_2.5_ levels were associated with increased FBG (Table 2). For an IQR increase in PM_2.5_ concentration in the previous 1-day (IQR = 5.73 μg/m^3^), 7-day (IQR = 4.25 μg/m^3^), and 28-day (IQR = 3.12 μg/m^3^), FBG increased 0.57 mg/dL [95% confidence inverval (CI): 0.02, 1.11, *p*-value = 0.04], 1.02 mg/dL (95% CI: 0.41, 1.63, *p*-value = 0.001), and 0.89 mg/dL (95% CI: 0.32, 1.47, *p*-value = 0.003), respectively. We also found associations of PM_2.5_ concentrations with IFG (i.e., FBG > 100 mg/dL), particularly for the longer moving averages of PM_2.5_ (Table 3). IQR increases in PM_2.5_ in the previous 1-day (IQR = 5.73 μg/m^3^), 7-day (IQR = 4.25 μg/m^3^) and 28-day (IQR = 3.12 μg/m^3^) exposure windows were associated with OR equal to 1.13 (95% CI: 0.97, 1.33, *p*-value = 0.12), 1.27 (95% CI: 1.06, 1.52, *p*-value = 0.01) and 1.32 (95% CI: 1.10, 1.58, *p*-value = 0.003) for IFG, respectively. We also obtained similar estimates when we used inverse probability weighting to reduce potential selection bias (see Table S2).

**Table 2:** Estimated change (and 95% CI) in fasting blood glucose level (mg/dL) per interquartile range increase in PM_2.5_ concentration averaged over the corresponding time window before each visit.

PM_2.5_ concentration	Participants *n*	Observations *n*	PM_2.5_ interquartile range (IQR)	Estimated change (95% CI) in FBG per IQR increase in PM_2.5_ concentrations	*p*-Value
1-day moving average	551	1,152	5.71 μg/m^3^	0.57 (0.02, 1.11)	0.04
7-day moving average	551	1,152	4.28 μg/m^3^	1.02 (0.41, 1.63)	0.001
28-day moving average	551	1,152	3.09 μg/m^3^	0.89 (0.32, 1.47)	0.003
Results from linear mixed-effects regression models accounting for correlation across multiple visits and adjusted for age, BMI, race, regular patterns of physical activity, smoking status, pack-years smoked, alcohol consumption, education level, statin use, temperature, and seasonality. Participants with diabetes were excluded.					

**Table 3:** Odds ratio (and 95% CI) of impaired fasting blood glucose (IFG) per interquartile range (IQR) increase in PM_2.5_ concentration averaged over the corresponding time window before each visit.

PM_2.5_ concentration	Participants *n*	Observations *n *	PM_2.5_ interquartile range (IQR)	Odds ratio (95% CI) of IFG per IQR increase in PM_2.5_ concentrations	*p*-Value
1-day moving average	551	1,152	5.73 μg/m^3^	1.13 (0.97, 1.33)	0.12
7-day moving average	551	1,152	4.25 μg/m^3^	1.27 (1.06, 1.52)	0.01
28-day moving average	551	1,152	3.12 μg/m^3^	1.32 (1.10, 1.58)	0.003
Note: IFG is defined as a fasting blood glucose level > 100mg/dL and < 126 mg/dL. Results from GEE models accounting for correlation across multiple visits and adjusted for age, BMI, race, regular patterns of physical activity, smoking status, pack-years smoked, alcohol consumption, education level, statin use, temperature, and seasonality. Participants with diabetes were excluded.					

### DNA Methylation and Mediation Analysis

**Figure 2:**
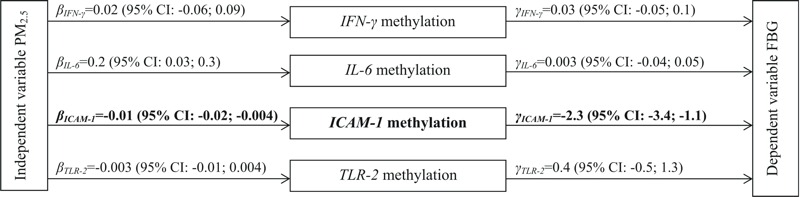
Inflammatory candidate gene methylation mediator model of the relationship between PM_2.5_ concentration and fasting blood glucose level. *ICAM-1* mean and *TLR-2* mean DNA methylation is log-normally distributed and *IL-6* and *IFN-*γ are normally distributed. β is the coefficient of the independent variable (PM_2.5_ 28-day moving average) when regressing the mediator (candidate gene methylation) on the independent variable, γ is the coefficient of the mediator when regressing the dependent variable (FBG) on both the independent variable and the mediator. Results from regression models are adjusted for age, BMI, race, regular patterns of physical activity, smoking status, pack-years smoked, alcohol consumption, education level, statin use, batch effects, percentage of lymphocytes, and percentage of neutrophils. Participants with diabetes were excluded.

PM_2.5_ showed a negative association with *ICAM-1* methylation, which, in turn, was negatively associated with FBG (Figure 2). Methylation of *IFN-*γ, *IL-6* and *TLR-2* showed no association with FBG. We also found no evidence of PM_2.5_-mediator interactions that changed FBG levels. Since a mediator needs to be associated with both the exposure and the outcome (Baron and Kenny 1986; Valeri and Vanderweele 2013), we conducted the analysis of mediation only for *ICAM-1* methylation. We examined the correlations between *ICAM-1* methylation and the other three genes and found no substantial correlations, which suggests that the separate analyses are fairly appropriate.

Before conducting the mediation analysis, we examined whether FBG levels at one visit (Y_ij_) affected *ICAM-1* methylation at the subsequent visit (M_ij + 1_) (i.e., FBG_ij_ ≥ *ICAM-1*_ij + 1_; dotted arrow in Figure 1), because it could potentially confound the mediator–outcome association and bias our estimates (Hernán et al. 2004). We found no association between Y_ij_ (FBG_ij_) and M_ij + 1_ (*ICAM-1*_ij + 1_). Point estimates were negligible (see Table S3). When we introduced a lag time and examined the effect of *ICAM-1* methylation on FBG levels in the subsequent visit (i.e., *ICAM-1*_ij_ ≥ FBG_ij + 1_), we also did not find an association (point estimate = –0.14, 95% CI: –1.93, 1.65).

Table 4 presents the natural direct effect, the natural indirect effect, and proportion mediated for *ICAM-1* methylation over the different PM_2.5_ moving averages. We fitted the exposure–mediator and the mediator–outcome model simultaneously and found substantial mediation effects of PM_2.5_ on FBG through a decrease in *ICAM* methylation for the 28-day exposure time window. The proportion mediated was larger (9%) for the 28-day exposure window, but small and negligible for the 1-day and 7-day moving averages.

**Table 4:** Mediation effect investigating whether blood *ICAM-1* methylation mediates the association between air pollution and fasting blood glucose level. Indirect effect represents the mediated effect through the *ICAM-1* methylation pathway.

PM_2.5_ concentration	Exposure to mediator association (β_PM2.5_)	Mediator to outcome association (γ_M_)	Mediated effect of *ICAM-1* methylation	Proportion mediated
1-day moving average	0.004 (–0.008, 0.008)	–2.65 (–4.41, –0.89)	–0.01 (–0.02, 0.004)	—^*a*^
7-day moving average	–0.0004 (–0.007, 0.006)	–2.69 (–4.45, –0.93)	0.001 (–0.02, 0.02)	1%
28-day moving average	–0.01 (–0.02, –0.004)	–2.47 (–4.23, –0.72)	0.03 (0.0001, 0.06)	9%
Note: Estimates correspond to 1-μg/m^3^ increase in PM_2.5_ concentration. Results from linear mixed-effects regression models accounting for correlation across multiple visits and adjusted for age, BMI, race, regular patterns of physical activity, smoking status, pack-years smoked, alcohol consumption, education level, statin use, temperature, seasonality, batch effect, % of lymphocytes, and % of neutrophils. Participants with diabetes were excluded. ^***a***^Proportion mediated cannot be estimated in this case because β_PM2.5_ and γ_M_ have opposite signs.				

We conducted further sensitivity analysis to examine if our results were robust to the no-unmeasured confounding assumptions required in the mediation analysis approach we used. Specifically, we excluded current smokers to limit residual confounding from smoking; we additionally controlled for total calorie intake and glycemic index to reduce potential confounding from diet; and we restricted the analysis to participants with a CRP level < 10 mg/L to partially remove effect from acute inflammation. Proportion mediated for *ICAM-1* methylation for the 28-day exposure time window was 9%, 7%, and 10%, respectively (see Table S4).

## Discussion

In the present study, we showed that PM_2.5_ concentrations estimated at the participants’ address were associated with higher FBG levels among nondiabetic individuals, as well as with higher odds of IFG. We also observed significant associations of lower blood *ICAM-1* methylation, which is expected to up-regulate the expression of ICAM-1 in blood leukocytes, with both higher PM_2.5_ levels and higher FBG levels.

Our study is consistent with previous epidemiology studies indicating that ambient PM is associated with metabolic dysregulation (Andersen et al. 2012; Brook et al. 2008; Eze et al. 2015; Puett et al. 2011; Rajagopalan and Brook 2012). Nevertheless, most previous studies either focused on T2DM or evaluated blood glucose over its entire range, including individuals with diabetes. The non-diabetic and pre-diabetic population represents an ideal target for primary prevention, which, however, has been understudied in air pollution research. Our findings of PM_2.5_ associations with FBG in this group may help identify individuals who are particularly susceptible to changes in FBG in the nondiabetic range. It is important to notice that the small changes in FBG resulted in significant odds of IFG, owing to the fact that many people are very close to 100 mg/dL in this older population, and a very small change in FBG may be enough to pass the threshold.

Our mediation analysis suggests that *ICAM-1* methylation in blood leukocytes served as a mediator of the association between PM_2.5_ and FBG, and we observed significant mediated effect at 28-day exposure time window. Our finding of higher concentrations of PM_2.5_ associated with lower methylation of the *ICAM-1* gene, which is expected to result in higher ICAM-1 expression, is consistent with previous literature indicating that elevated concentrations of PM are associated with an increase in expression of endothelial markers (Bind et al. 2012; Madrigano et al. 2010; O’Neill et al. 2007; Rückerl et al. 2006). The ICAM-1 glycoprotein is responsible for leukocyte adhesion, homing, and transmigration during inflammatory responses (Rahman and Fazal 2009). Exposure to PM_2.5_ may cause local inflammation in the lungs and promote circulating leukocytes in blood to transmigrate to the target tissue through the up-regulation of adhesion molecules on the endothelial cell surface. Recent observational and intervention studies have linked elevated concentrations of plasma endothelial adhesion molecules with markers of insulin resistance and increased risk of T2DM (Blüher et al. 2002; Hak et al. 2001; Meigs et al. 2004). The ICAM-1 glycoprotein may facilitate migration of leukocytes from the blood to the adipose tissue (Mendez et al. 2013; Rao et al. 2015; Sun et al. 2009), which could result in local inflammation and subsequently insulin resistance. Alternatively, the ICAM-1 glycoprotein may also facilitate leukocyte transmigration to the pancreas (Yagi et al. 1995), which could affect beta-cell function and result in impaired insulin secretion. Although the observed association was relatively modest, our estimates were comparable to many other studies evaluating the association between ambient air pollution and DNA methylation with similar exposure levels (Guo et al. 2014; Madrigano et al. 2011). DNA methylation is measured as a percentage, which indicates the proportion of cells, or more accurately of haploid genomes, which show methylation at the sequence being analyzed. The differences in DNA methylation reported in our study are related to the presence upon PM_2.5_ exposure of higher numbers of circulating blood cells within no methylation at the *ICAM-1* promoter. Further research is needed to determine whether these cells correspond to a specific leukocyte population with known function and their potential roles in relation to PM_2.5_ effects.

Conversely, methylation on the cytokine genes investigated (i.e., *IFN-*γ and *IL-6*) was not implicated as a mediator in this study. Many pro- and anti-inflammatory cytokines act in concert to trigger the inflammatory cascade. Future research may expand the number of inflammatory cytokines investigated and examine their joint effects.

One interesting aspect of the human methylome is that it exhibits both dynamic and static patterns. For instance, methylation of imprinted genes and genes drives tissue lineage commitment and differentiation that is established during embryogenesis and persists through life (Li et al. 1993); on the other hand, DNA methylation of inflammatory genes may change rapidly after environmental insults (Guo et al. 2014; Hou et al. 2014; Tarantini et al. 2009). Our results are consistent with the dynamic nature of methylation levels in inflammatory pathways, which allows for fine tuning of inflammatory responses. Nevertheless, we are aware that differences in DNA methylation do not necessarily translate into gene expression changes. DNA methylation is only one of the regulatory machineries that control gene expression. Other regulatory mechanisms, such a transcription factor activation, histone modification, chromatin remodeling and RNA silencing may also contribute to regulation of gene expression at various stages.

This study has a number of strengths. We estimated concentrations of PM_2.5_ at the residential address for each participant using a state-of-art hybrid model. Estimates from this hybrid model serve as better surrogates relative to the standard use of data from monitoring stations for each participant’s actual exposure, and limit exposure misclassifications. We conducted analyses both on FBG as a continuous variable and on IFG, a dichotomized variable constructed using a well-established cutoff for preclinical alterations in glucose metabolism. These two sets of analyses produced highly consistent results. We used repeated measures, which were accounted for using linear mixed-effects models with subject specific intercepts for FBG and generalized linear equations (GEE) model with empirical variance for IFG. We conducted mediation analysis as a novel approach for DNA methylation studies. This approach is particularly useful for investigating effects due to environmental exposures. While Mendelian randomization—a method that relies on genotype data used as instrumental variables—is often proposed to identifying epigenetic mediation, this approach cannot be used for external risk factors, such as PM, which, due to their nature, are not associated with the participants’ genetic sequences (such as single nucleotide polymorphisms) (Relton and Davey Smith 2012). Finally, we measured DNA methylation in candidate genes by pyrosequencing, which yields high precision (Tost and Gut 2007).

Our study has a few notable limitations. We focused on correlations among DNA methylation and FBG at the same clinical visit. It is therefore difficult to disentangle the temporal relationship between DNA methylation and FBG concentrations. However, when we examined the effect of DNA methylation on FBG for the subsequent visit (i.e., *ICAM-1*_ij_ ≥ FBG_ij + 1_), we did not find any association. We also found no association of FBG on *ICAM-1* methylation at the subsequent visit (i.e., FBG_ij_ ≥ *ICAM-1*_ij + 1_). These analyses confirm that the effects we observed represent short- and medium-term responses to PM_2.5_ and are not persistent over the 3–5 years between the medical visits. To obtain valid estimates for the natural indirect effects, we made the following assumptions: *a*) no-unmeasured confounding between PM_2.5_ concentration and FBG levels, *b*) no-unmeasured confounding between PM_2.5_ concentration and methylation, *c*) no-unmeasured confounding between methylation and FBG levels, and *d*) no methylation-FBG confounders affected by the exposure. We conducted a number of sensitivity analyses to test the robustness to the no-unmeasured confounding assumptions. We excluded participants who were current smokers to better control for residual confounding by smoking; we additionally controlled for total calorie intake and glycemic index, to limit potential confounding from diet; we also restricted the analysis to participants with a CRP level < 10 mg/L, to partially remove potential effect from acute inflammation. In addition, we assessed the validity of assumption *d* either based on subject knowledge or empirically (Bind et al. 2016). From previous subject knowledge, we assumed that PM_2.5_ concentration would not affect participants’ age, race, smoking status, and statin use, as well as the batch of methylation measurements and seasonality. We also tested whether PM_2.5_ concentration would influence participant’s BMI, regular patterns of physical activity, percentage of lymphocytes, and percentage of neutrophils, by regressing PM_2.5_ concentration on each of these potential confounders. None of the above mediator–outcome confounders in current analysis were affected by the exposure (*p*-values were 0.10, 0.54, 0.50, and 0.78, respectively). Another limitation of our study is the potential for measurement error in both the exposure and FBG. However, we expect both measurement errors to be nondifferential and therefore to attenuate—rather than to cause—the observed significant associations.

## Conclusion

In conclusion, we found that PM_2.5_ concentrations are associated with higher FBG level, and this association was in part mediated through *ICAM-1* gene methylation, particularly at the longer (28-day) moving average investigated. Our study demonstrates a novel approach of mediation analysis in epigenetic studies and highlights a mediating role of *ICAM-1* gene methylation in air pollution–associated abnormal glucose metabolism. While the proportion mediated by *ICAM-1* methylation alone is relatively modest, methylation of other genes not investigated in this study, independently or in combination with *ICAM-1* methylation, may mediate larger proportions of PM_2.5_ effects. Future epigenome-wide studies are needed to determine the extent to which DNA methylation contributes to mediate environmental effects on human metabolism.

## Supplemental Material

(1.3 MB) PDF
