# Supplementary material for: Particulate Air Pollution and Fasting Blood Glucose in Nondiabetic Individuals: Associations and Epigenetic Mediation in the Normative Aging Study, 2000–2011
Source: Environ Health Perspect. 2016 May 26;124(11):1715–21. doi: 10.1289/EHP183 (PMC5089881; doi:10.1289/EHP183)

Environ Health Perspect
DOI: 10.1289/EHP183

Note to readers with disabilities: *EHP* strives to ensure that all journal content is accessible to all readers. However, some figures and Supplemental Material published in *EHP* articles may not conform to 508 standards due to the complexity of the information being presented. If you need assistance accessing journal content, please contact ehp508@niehs.nih.gov. Our staff will work with you to assess and meet your accessibility needs within 3 working days.

Supplemental Material

Particulate Air Pollution and Fasting Blood Glucose in Non-Diabetic Individuals: Associations and Epigenetic Mediation in the Normative Aging Study, 2000-2011

Cheng Peng, Marie-Abele C. Bind, Elena Colicino, Itai Kloog, Hyang-Min Byun, Laura Cantone, Letizia Trevisi, Jia Zhong, Kasey Brennan, Alexandra E. Dereix, Pantel S. Vokonas, Brent A. Coull, Joel D. Schwartz, and Andrea A. Baccarelli

Table of Contents

Table S1. Summary statistics of $PM_{2.5}$ (PM with aerodynamic diameter ≤2.5 µm) and temperature levels.

Table S2. Estimated change (and 95% CI) in fasting blood glucose (FBG) level (mg/dL) per interquartile range (IQR) increase in $PM_{2.5}$ (particulate matter with aerodynamic diameter ≤2.5 µm) concentration averaged over the corresponding time window before each visit. We accounted for potential selection bias using inverse probability weighting.

Table S3. Estimated change (and 95% CI) in inflammatory candidate gene methylation with increase in fasting blood glucose [FBG (mg/dL)] concentrations at previous visits ($Y_{ij} \rightarrow M_{ij+1}$)

Table S4. Sensitivity analysis of the mediation effect of *ICAM-1* methylation on the association between $PM_{2.5}$ concentrations averaged over 28-day exposure window with fasting blood glucose (FBG) level (mg/dL). Natural indirect effect represents the “mediated” effect through the *ICAM-1* methylation pathway. Estimates correspond to 1 µg/m^3 increase in $PM_{2.5}$ concentration.

Figure S1. Methylation of specific CpG sites for the four candidate genes (interferon gamma (*IFN-γ*), interleukin-6 (*IL-6*), Toll-like receptor 2 (*TLR-2*), and intracellular adhesion molecule-1 (*ICAM-1*)).

Figure S2. Timeline of the study design.

Table S1. Summary statistics of $PM_{2.5}$ (PM with aerodynamic diameter ≤2.5 μm) and temperature levels. $PM_{2.5}$ and temperature were summarized as cumulative averaged exposures up to the previous 28-day exposure window during the study period. $PM_{2.5}$ was estimated using spatiotemporal land-use regression models estimating levels at the participants' residential addresses. Temperature values were obtained through the national climatic data center (NCDC); grid cells were matched to the closest weather station for meteorological variables.

	**Mean**	**SD**	**Percentiles**				
			**10th**	**25th**	**50th**	**75th**	**90th**
**$PM_{2.5}$ (μg/m^3)**							
1-day moving average	10.92	5.42	5.86	7.37	9.47	13.08	17.54
7-day moving average	10.59	3.48	6.75	8.09	10.06	12.37	14.79
28-day moving average	10.71	2.62	7.57	8.94	10.45	12.03	13.89
**Temperature (^{o}C)**							
1-day moving average	11.93	7.59	1.92	6.24	12.11	18.00	21.80
7-day moving average	11.80	7.30	1.41	6.26	12.42	18.16	20.87
28-day moving average	11.85	7.14	1.54	5.86	12.82	18.38	20.52

Table S2. Estimated change (and 95% CI) in fasting blood glucose (FBG) level (mg/dL) per interquartile range (IQR) increase in $PM_{2.5}$ (particulate matter with aerodynamic diameter ≤2.5 μm) concentration averaged over the corresponding time window before each visit. We accounted for potential selection bias using inverse probability weighting.

$PM_{2.5}$ concentration	N of participants	N of observations	$PM_{2.5}$ Interquartile Range (IQR)	Estimated change (95% CI) in FBG per IQR increase in $PM_{2.5}$ concentrations
1-day moving average	551	1133	5.71 μg/m^3	0.51 (-0.04; 1.05)
7-day moving average	551	1133	4.28 μg/m^3	0.91 (0.29; 1.53)
28-day moving average	551	1133	3.09 μg/m^3	0.80 (0.21; 1.39)

Results from linear mixed-effects regression models accounting for correlation across multiple visits and adjusted for age, BMI, race, regular patterns of physical activity, smoking status, pack-years smoked, alcohol consumption, education level, statin use, temperature, and seasonality. Participants with diabetes were excluded.

Potential selection bias due to loss to follow-up was accounted for using inverse probability weighting. In a logistic regression, we predict the probability of coming to a subsequent visit by covariates from the previous one, which include age, BMI, regular patterns of physical activity, smoking status, pack year smoked, FEV1 and FVC ratio, medication (diuretics and beta blocker), and education level.

Table S3. Estimated change (and 95% CI) in inflammatory candidate gene methylation with increase in fasting blood glucose [FBG (mg/dL)] concentrations at previous visits ($Y_{ij} \rightarrow M_{ij+1}$).

Candidate gene methylation	N of subjects	Estimated change (95% CI) in $M^{i,j=J+1}$ per 1 mg/dL increase in $Y^{i,j=J}$
IFN-γ	472	-0.02 (-0.05; 0.02)
IFN-γ adjusted for $M^{i,j=J}$	463	-0.02 (-0.06; 0.01)
IL-6	472	-0.04 (-0.11; 0.04)
IL-6 adjusted for $M^{i,j=J}$	466	-0.03 (-0.09; 0.02)
*ICAM-1**	472	0.001 (-0.002; 0.004)
*ICAM-1** adjusted for $M^{i,j=J}$	424	0.001 (-0.002; 0.004)
*TLR-2**	472	0.003 (-0.002; 0.007)
*TLR-2** adjusted for $M^{i,j=J}$	415	0.003 (-0.002; 0.008)

Results from linear mixed-effects regression models accounting for correlation across multiple visits, and adjusted for age, BMI, race, regular patterns of physical activity, smoking status, pack-years smoked, alcohol consumption, education level, statin use, batch effects, percentage of lymphocytes, and percentage of neutrophils (all from previous visits). Participants with diabetes were excluded.

**ICAM-1* and *TLR-2* DNA methylation measurements were log transformed.

Table S4. Sensitivity analysis of the mediation effect of *ICAM-1* methylation on the association between $PM_{2.5}$ concentrations averaged over 28-day exposure window with fasting blood glucose (FBG) level (mg/dL). Natural indirect effect represents the "mediated" effect through the *ICAM-1* methylation pathway. Estimates correspond to 1 $\mu g/m^3$ increase in $PM_{2.5}$ concentration.

**$PM_{2.5}$ concentration averaged over 28-day moving average**	**Exposure to mediator association ($\beta_{PM2.5}$) (95% CI)**	**Mediator to outcome association (γ_M) (95% CI)**	**Natural indirect effect of *ICAM-1* methylation (95% CI)**	**Proportion mediated**
Model 1[a]	-0.01 (-0.02; -0.006)	-2.30 (-3.43; -1.17)	0.03 (-0.002; 0.06)	9%
Model 2[b]	-0.01 (-0.02; -0.004)	-2.32 (-4.59; -0.05)	0.03 (-007; 0.07)	7%
Model 3[c]	-0.01 (-0.02; -0.005)	-2.99 (-4.13; -1.85)	0.03 (0.0006; 0.07)	10%

Results from linear mixed-effects regression models adjusted for age, BMI, race, regular patterns of physical activity, smoking status, pack-years smoked, alcohol consumption, education level, statin use, temperature, seasonality, batch effect, percentage of lymphocytes, percentage of neutrophils. Participants with diabetes were excluded.

[a] We excluded current smokers to limit potential residual confounding by smoking.

[b] We additionally controlled for dietary intake (total calorie intake and glycemic index), to reduce potential confounding by diet.

[c] We restricted the analysis to participants with a C-reactive protein (CRP) level less than 10 mg/L, to partially removal potential effect from acute inflammation.

Figure S1. Methylation of specific CpG sites for the four candidate genes (interferon gamma (*IFN-γ*), interleukin-6 (*IL-6*), Toll-like receptor 2 (*TLR-2*), and intracellular adhesion molecule-1 (*ICAM-1*)).

i. *ICAM-1* methylation CpG sites

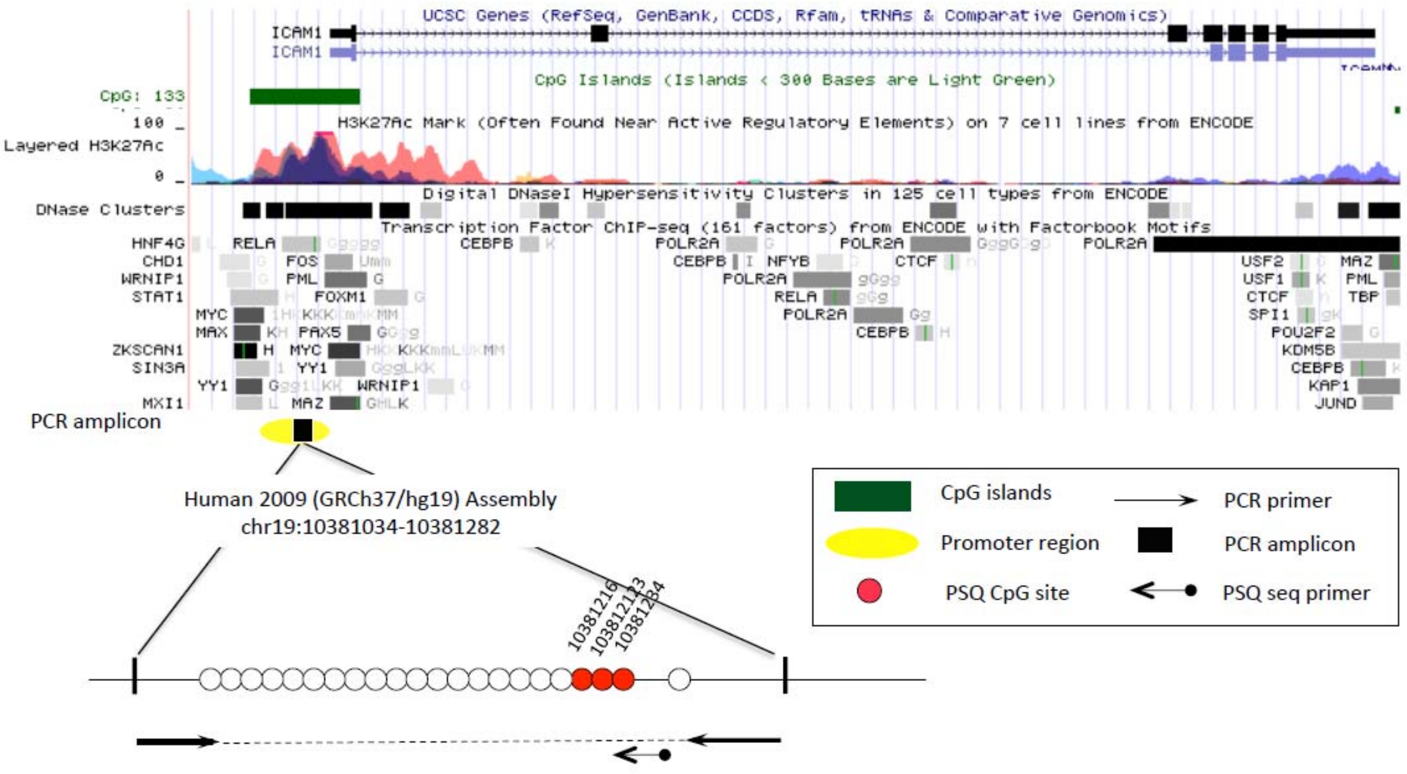

Note: PCR: polymerase chain reaction; PSQ: pyrosequencing; PSQ CpG sites are the methylation sites where pyrosequencing was performed.

ii. *IFN-γ* methylation CpG sites

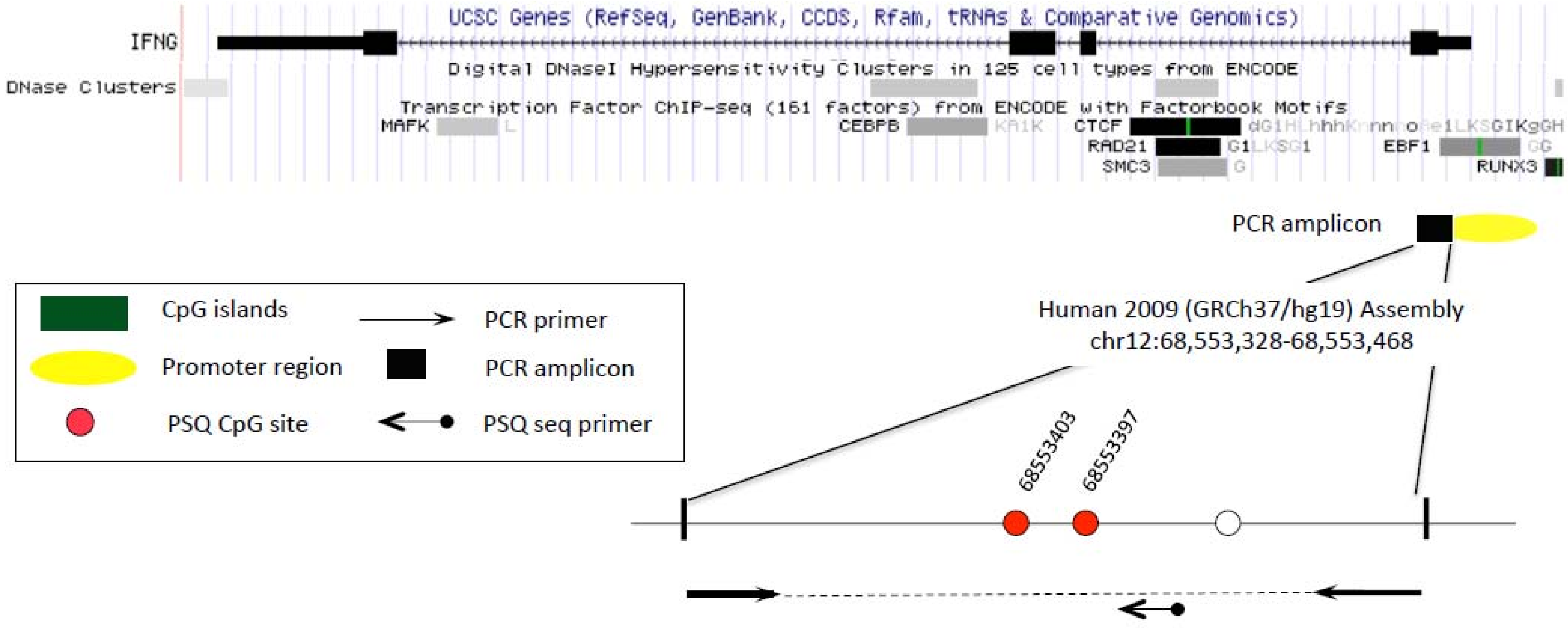

iii. *IL-6* methylation CpG sites

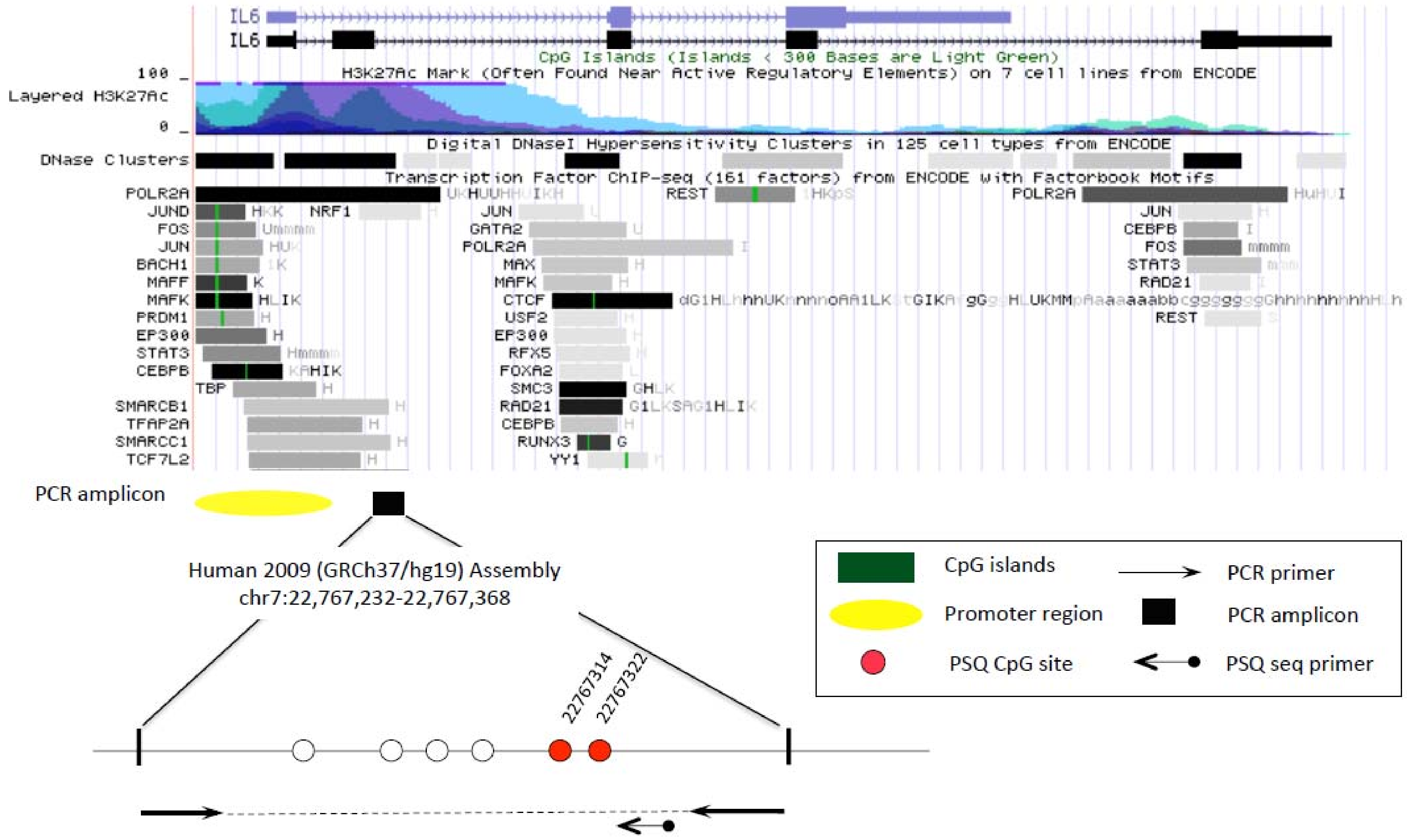

iv. *TLR-2* methylation CpG sites

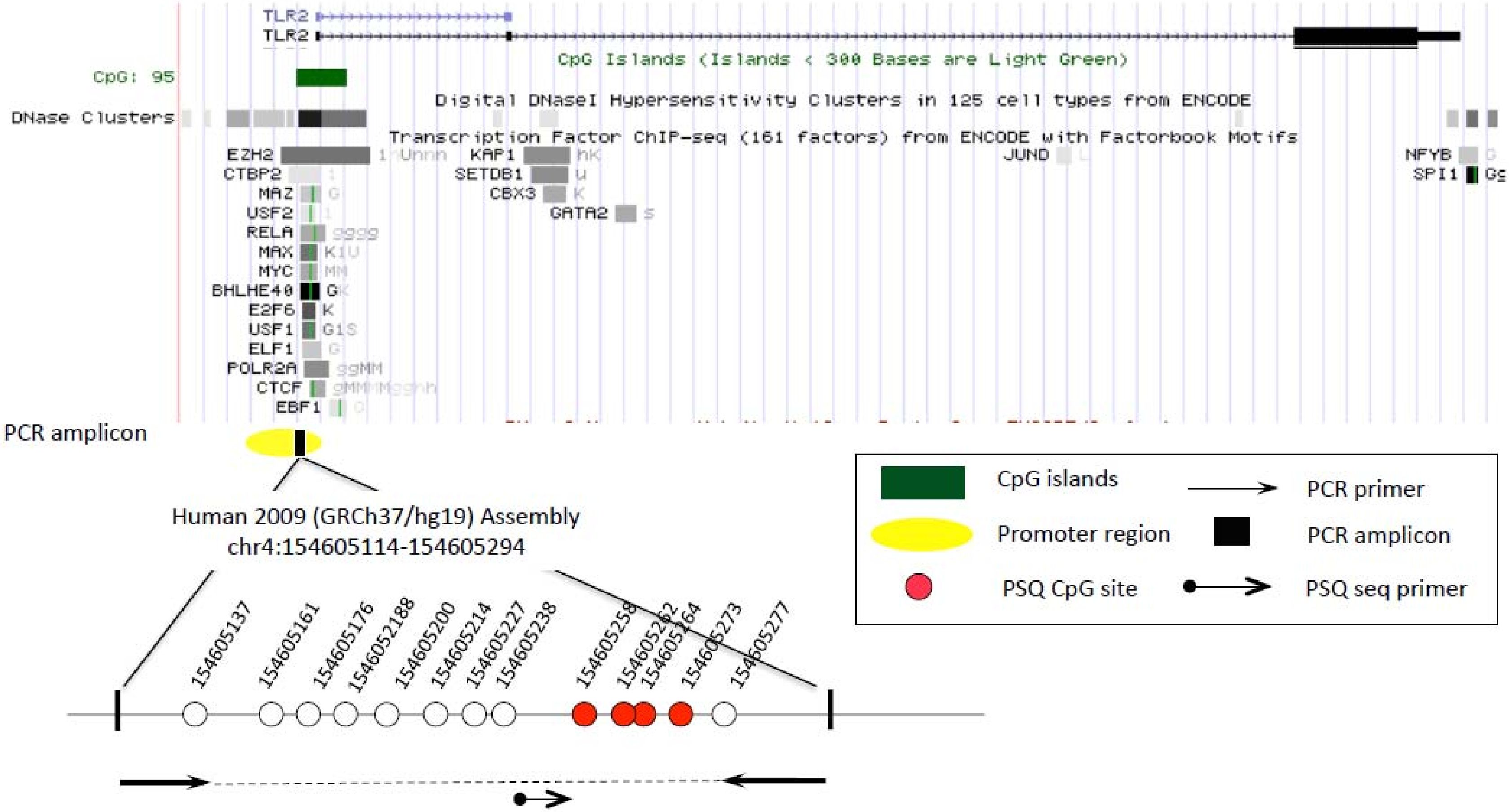

Figure S2. Timeline of the study design.

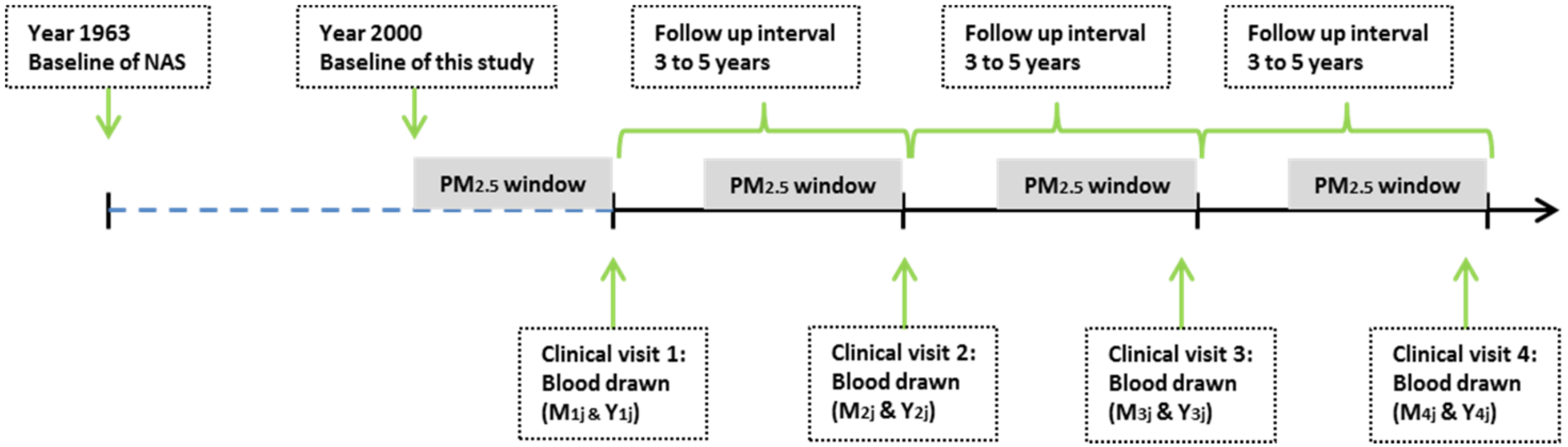

Supplement: (1.3 MB) PDF [file EHP183.s001.acco.pdf]
